# No Time to Dye: Portable Surface-Enhanced Raman Scattering for On-Site Detection of Sudan IV Dye in Palm Oil

**DOI:** 10.1021/acsomega.6c06638

**Published:** 2026-10-08

**Authors:** Joe Stradling, Amy Colleran, Nigel Gotts, Yun Xu, James Simons, Wilfred A. Abia, Howbeer Muhamadali, Royston Goodacre

**Affiliations:** † Centre for Metabolomics Research, Department of Biochemistry, Cell and Systems Biology, Institute of Systems, Molecular and Integrative Biology, 4591 University of Liverpool, Liverpool L69 7ZB, U.K.; ‡ Institute for Global Food Security, School of Biological Sciences, Queens University Belfast, 19 Chlorine Gardens, Belfast BT9 5DL, U.K.; § Laboratory of Pharmacology and Toxicology, Department of Biochemistry, Faculty of Science, University of Yaounde 1, Yaounde AEFAS 812, Cameroon

## Abstract

The widespread use of Sudan IV dye as an adulterant in palm oil poses significant public health risks. This nonfood-grade dye, linked to carcinogenic effects, has prompted a pressing need for efficient, rapid, and reliable field-portable detection methods. This study investigates the application of hand-held surface-enhanced Raman scattering (SERS) technology for the rapid and sensitive detection of Sudan IV dye in palm oil. By leveraging the enhanced sensitivity of SERS, we provide a field-deployable solution that can be utilized in local markets and production sites, facilitating real-time monitoring and ensuring food safety. Using a univariate calibration approach, a predictive model was developed to quantify Sudan IV in palm oil, achieving a limit of detection (LOD) of 0.0065 mg/mL. In total, 64 palm oil samples obtained from markets in Cameroon and 9 palm oil samples purchased online were screened for Sudan IV using SERS, which identified four online samples as testing positive for this azo dye. To verify these findings, liquid chromatography–mass spectrometry (LC-MS) was subsequently performed on the samples bought online. LC-MS analysis confirmed the presence of Sudan IV in the four SERS-positive oils purchased from an online retailer and additionally detected the dye in a fifth oil from the same source.

## Graphical abstract

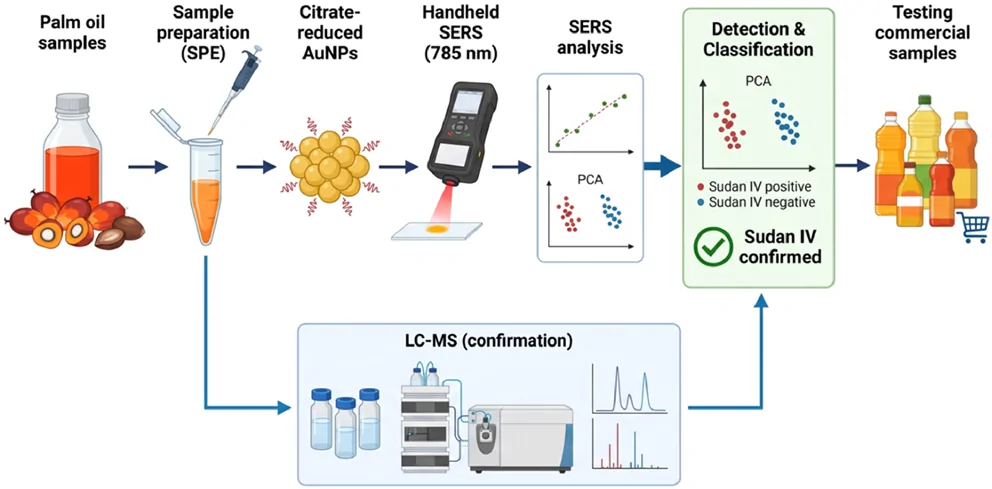

## Introduction

Palm oil naturally ranges in color from pale orange to deep red, a variation largely driven by its differing levels of β-carotene.^1,2^ A deeper red color is generally associated with higher levels of carotenoids, which are nutritionally beneficial compounds, and is therefore often used as an indicator of higher perceived quality and value in addition to increased market price.^3,4^ However, this same visual quality marker also creates a vulnerability to economic adulteration. Because color intensity is strongly associated with value, palm oil can be deliberately colored with illegal red dye to mimic the appearance of high-carotenoid oil or to compensate for bleaching and refining processes that remove natural pigments.

Sudan dyes are synthetic azo compounds widely employed in various industrial applications, such as textiles, plastics, and printing due to their vibrant color properties. When added to palm oil, Sudan IV intensifies the red coloration of the oil, making it appear more desirable to consumers, and is frequently reported as the predominant adulterant in palm oil fraud cases, likely due to its higher solubility in crude palm oil compared with Sudan I, Sudan II, and Sudan III, which enables more effective coloration.^5–7^ The potential carcinogenicity of Sudan IV presents a substantial health concern when used as a food colorant, prompting many regulatory authorities to prohibit its use in food products.^8,9^ This concern is underscored by regulatory surveillance data, which indicate that of 212 documented cases of Sudan dye adulteration in palm oil between 2004 and 2022, a striking 81% (171 cases) occurred in Africa.^7^ The persistent use of these harmful additives highlights the urgent need for effective monitoring and regulation to safeguard public health.

The detection of Sudan IV dye in palm oil is challenging for numerous reasons, primarily due to the low concentrations at which the dye is typically present and the complex nature of the hydrophobic oil matrix. Traditional analytical methods such as high-performance liquid chromatography (HPLC) and mass spectrometry (MS) have been employed to detect these contaminants with high accuracy.^10,11^ However, these techniques are not without limitations as they are time-consuming, expensive, require sophisticated laboratory-based equipment, and lengthy sample preparation making them impractical for routine, on-site testing in resource-limited settings.

Given these challenges, there is a pressing need for portable, cost-effective, rapid, and sensitive detection methods that can be used directly in the field to monitor Sudan IV dye contamination in palm oil.^12^ SERS presents a promising solution to this problem as it is an advanced analytical technique that significantly enhances the Raman scattering signals of target molecules, thereby enabling the detection of trace amounts of contaminants.^13,14^ The portability and sensitivity of SERS technology make it an ideal candidate for fast real-time, on-site testing, offering a practical tool for regulatory authorities and food safety agencies.^15^

Several studies have explored the use of SERS for detecting Sudan dyes, ranging from demonstrating its effectiveness by spiking organic solvents with the dye, to validating the method as a high-throughput, low-cost, and reliable technique, as well as optimizing the extraction of the dyes from food matrices.^16–19^ These studies have also focused on optimizing dye extraction techniques from complex food matrices like palm oil, showcasing the method’s versatility and sensitivity in practical applications.^19^ In contrast, the present study evaluates the use of a commercially available hand-held Raman/SERS device for the rapid screening of palm oil samples obtained directly from Cameroonian markets and online UK suppliers, with the analytical findings subsequently validated using high-resolution Orbitrap LC-MS analysis on the same sample set. By integrating portable spectroscopy with confirmatory mass spectrometry, this work assesses the feasibility of a rapid, field-deployable screening approach for palm oil authentication and food safety monitoring.

## Experimental Methods

### Synthesis of Citrate-Stabilized Gold Nanoparticles (cAuNPs)

Citrate-stabilized gold nanoparticles were synthesized using the Lee and Meisel method.^20^ Chloroauric acid (0.24 g) was dissolved into 500 mL of deionized water and brought to the boil, with continuous stirring. Trisodium citrate (1.0 g) was dissolved in 100 mL of deionized water and 50 mL of trisodium citrate solution was added dropwise, slowly to the chloroauric acid/water solution. The solution was left to boil for 1 h and then left to return to room temperature. A change in color of the solution was observed from yellow to a dark red/purple indicating successful nanoparticle formation.

The SERS nanoparticles were characterized by scanning electron microscopy (SEM) for particle size estimation, while surface charge (ζ-potential) and hydrodynamic diameter were determined using a Zetasizer (Supporting Information Figures S1–S3).

### Palm Oil Collection

Oils A-I were purchased from an online UK supplier. Sixty-four red palm oil samples (samples 1–64) were purchased from retailers in open markets in the two metropolitan cities in Cameroon, notably, Yaounde (administrative capital) and Douala (economic capital). A cluster sampling technique was used, with each palm oil sample collected randomly. Each sample was collected in 1 L plastic bottle and transported at room temperature to the laboratory and stored at room temperature until analysis.

### Solid Phase Extraction (SPE)

SPE was performed according to Genualdi *et al*., under vacuum to facilitate the elution process.^21^ For the extraction, 0.5 mL of each palm oil sample was mixed with 1 mL of hexane and vortexed for 1 min. For each extraction a 6 mL Supelclean LC-Alumina-B SPE Tube (Merck) was used. The cartridge was first conditioned with 6 mL of methanol, 6 mL of ethyl acetate, and 6 mL of hexane. The sample was added to the cartridge and washed with 6 mL of hexane, 6 mL of diethyl ether, and finally 2 mL of ethyl acetate. The Sudan dyes were then eluted from the column using 8 mL of ethyl acetate and methanol (90:10, v/v). The extract was then dried using a Savant SpeedVac (SPD130DLX). The samples were randomly divided into three groups to enable spectral acquisition on three separate days.

### SERS Measurements

Prior to spectral acquisition, the dried extract was reconstituted in 0.2 mL of methanol. The SERS spectra were obtained using a SnRI CBeX (Snowy Range, Laramie, Wyoming) portable Raman spectrometer in vial mode. A laser wavelength of 785 nm was used for illumination for an acquisition time of 1 s at a power of 50 ± 3 mW on the sample. The spectral range was 400–2300 cm^–1^ with one data-point per wavenumber. For SERS analysis the reconstituted extract was mixed with 0.4 mL of nanoparticles. This was vortexed for 5 s followed by the addition of 0.05 mL of NaCl (0.25 M) and vortexed for 5 more seconds. The vial containing the mixture was then immediately inserted into the spectrometer holder ready for analysis. Supporting Information Figure S4 demonstrates a confirmation of the peaks which can be attributed to Sudan IV dye.

### LC-MS Measurements

LC-MS analysis of the samples was undertaken using a Thermo Scientific system, comprising a Vanquish LC stack (including a UV/vis Diode Array Detector), and an Orbitrap ID-X MS (operating in ESI positive ion mode with Orbitrap resolution at 120 k for MS scan). The LC method applied was targeted at lipids, utilizing a Thermo Scientific Hypersil Gold column (100 × 2.1 mm, 1.9 μm particle size, part number 25002-102130) at 55 °C, with a solvent gradient of 0.4 mL/min, with solvent A of 60/40 acetonitrile/water plus 10 mM ammonium formate and 0.1% Formic Acid, and solvent B of 85.5/9.5/5 isopropanol/acetonitrile/water plus 10 mM ammonium formate and 0.1% formic Acid. The Thermo Scientific Vanquish Diode Array Detector (VF-D11-A) was set to monitor channels 525 and 357 nm (for Sudan IV), plus a 3D Field of 300–600 nm. Data acquisition involved injections of each sample being separated from that of the next sample by two blank (methanol) injections. Results were plotted using Thermo Scientific’s Freestyle application within their Xcalibur software.

### Data Analysis

All SERS data were processed using MATLAB R2024a (The MathWorks, Natick, MA). For principal component analysis (PCA), the spectra were first subjected to vector normalization. To build the univariate prediction model a local baseline correction in the spectral region between 1072 and 1119 cm^–1^ was applied to both blank and sample spectra by subtracting a linear baseline connecting the start and end points of the diagnostic Sudan IV peak. The standard deviation of the baseline-corrected blanks was used to estimate measurement noise. For each sample, the peak height at the defined apex was extracted and used to construct a linear calibration model relating peak height to Sudan IV concentration. To determine the limit of detection (LOD), the following equation was applied after baseline correction using data from the five lowest Sudan IV spiking concentrations, with the blanks (not spiked with dye) omitted from the calculation

1 $$LOD=\frac{\left(3\times SD\,\;of\,\;blank\right)\pm C}{m}$$

where SD is the standard deviation, *C* is the *y*-intercept and *m* is the slope.^22^

## Results and Discussion

### Correlation Between Sudan IV Concentration and SERS Response

Sudan IV dye was spiked into a palm oil sample that did not contain the dye (confirmed using LC-MS) at concentrations between 0.005 and 0.075 mg/mL in increments of 0.005 mg/mL, including a control palm oil sample with no adulteration (also confirmed using LC-MS). Following this, these 15 spiked samples, plus control, were put through solid phase extraction (SPE) and then prepared for SERS analysis. In total, 5 samples from each concentration were extracted, followed by 3 SERS measurements for each of the 5 samples, which gives a total of 15 spectra per concentration. The SERS spectra of Sudan IV display distinct bands at 1096, 1142, 1198, 1294, and 1425 cm^–1^ (Figure 1), which are consistent with vibrational modes associated with the azo (N=N) linkage, C–N stretching, and aromatic ring vibrations characteristic of Sudan IV.^23,24^ It is evident that these vibrational features increase in intensity with increasing dye concentration, reflecting a greater likelihood of Sudan IV molecules occupying SERS hotspots.

**Figure 1:**
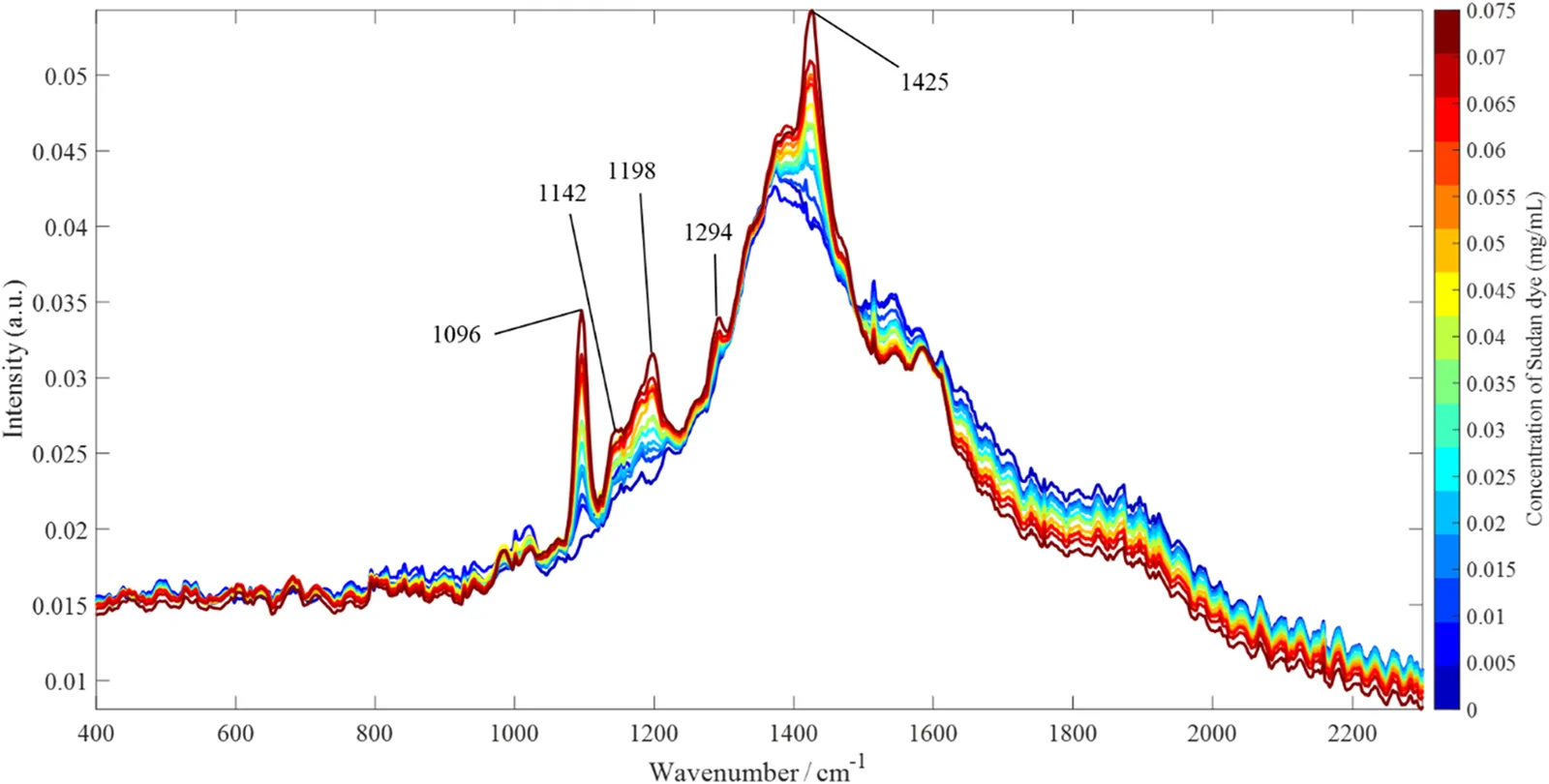
Normalized SERS spectra of palm oil spiked between 0 and 0.075 mg/mL of Sudan IV dye.

Principal Component Analysis (PCA) is an effective multivariate technique for observing the relationship between samples, and this exploratory multivariate method is an excellent approach for visualizing and interpreting variations within complex spectroscopic data sets such as SERS spectra.^25,26^ The PCA scores plot (Figure 2a) of the SERS spectra acquired at increasing dye concentrations exhibits a clear trend along the PC-1 axis, with samples progressing from left to right as the concentration of the spiked Sudan IV increases. This systematic separation indicates that PC-1 is strongly correlated with dye concentration, which accounted for 88.87% of the total explained variance (TEV) in the data. Examination of the PC-1 loading plot (Figure 2b) reveals that the bands contributing most significantly to this variance correspond to the 5 characteristic vibrational modes of Sudan IV detailed above, confirming that changes in dye-related spectral features dominate the observed variance.

**Figure 2:**
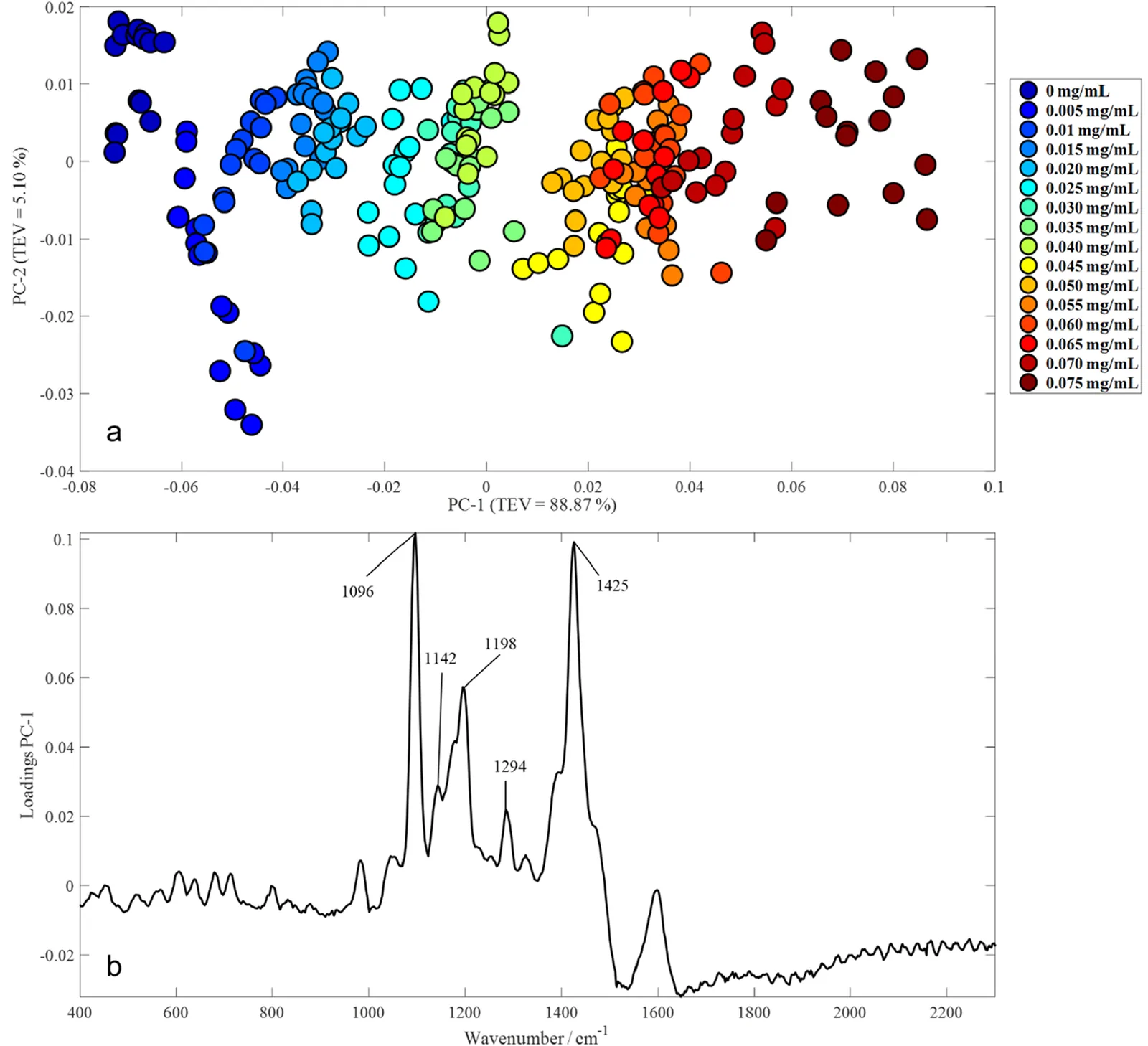
PCA scores plot of SERS data normalized along with the total explained variance (TEV) of the first two principal components (a), as well as the associated PC-1 loading spectrum (b).

### SERS Applied to Oils Collected from Cameroon

The adulteration of palm oil with Sudan IV continues to be a significant and widespread problem in markets across Africa and Asia. To evaluate the applicability of hand-held SERS for detecting this illegal dye under real-world conditions, a total of 64 authentic palm oil samples were collected by W.A. from markets throughout Cameroon. Each sample was subjected to the same extraction protocol and SERS measurement procedure used for the laboratory-prepared, intentionally spiked oils. Three separate aliquots of each sample were processed, with one SERS measurement taken from each extraction. Comparison of the resulting spectra (Figure 3) revealed that the Cameroonian samples lacked the characteristic Sudan IV peaks present in the spiked oils, indicating that no detectable levels of the dye were present in the samples analyzed.

**Figure 3:**
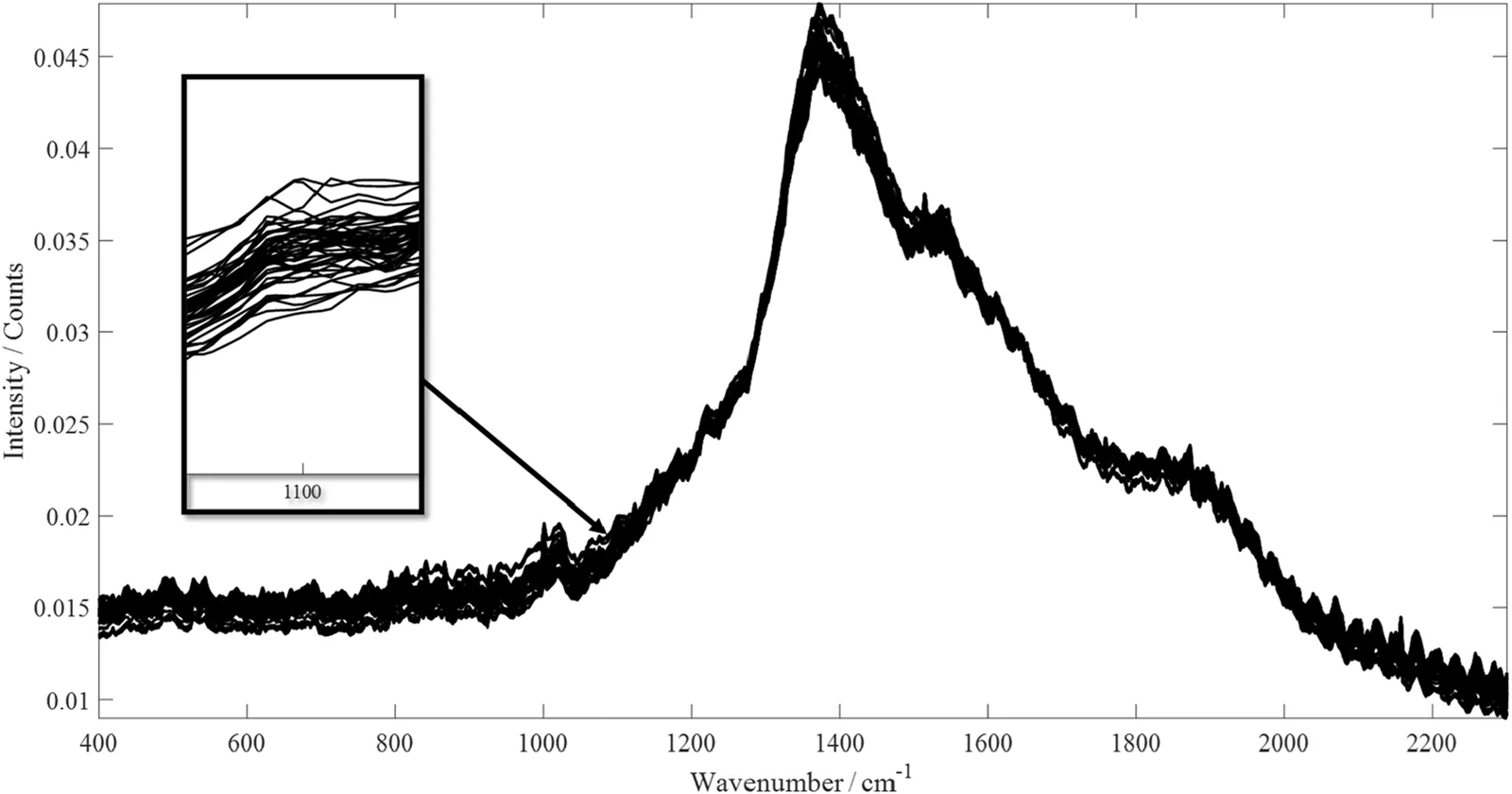
Normalized SERS spectra of samples 1 to 64 which were purchased from markets in Cameroon. A total of three spectra were recorded from each sample and then averaged.

### SERS Applied to Store Bought Samples

To expand the assessment of potential adulteration, further commercial samples were sourced from an online UK retailer. In total, 9 bottles of palm oil were purchased from the online retailer (labeled A to I); visible images of a subset of these oils are shown in Supporting Information Figure S5. Three aliquots were taken from each bottle and subjected to SPE, after which a single SERS measurement was recorded for each extract. Comparison of these spectra (Figure 4) with those from the intentionally spiked palm oil samples (Figure 1) revealed several notable similarities. Sample B exhibited strong bands corresponding to the Sudan IV dye, while sample I also showed features in the same spectral regions, albeit with lower intensity. Samples C and D displayed a distinct band at 1096 cm^–1^, indicating the possible presence of the dye; however, these samples showed weak or negligible intensity at the other expected characteristic Sudan dye bands.

**Figure 4:**
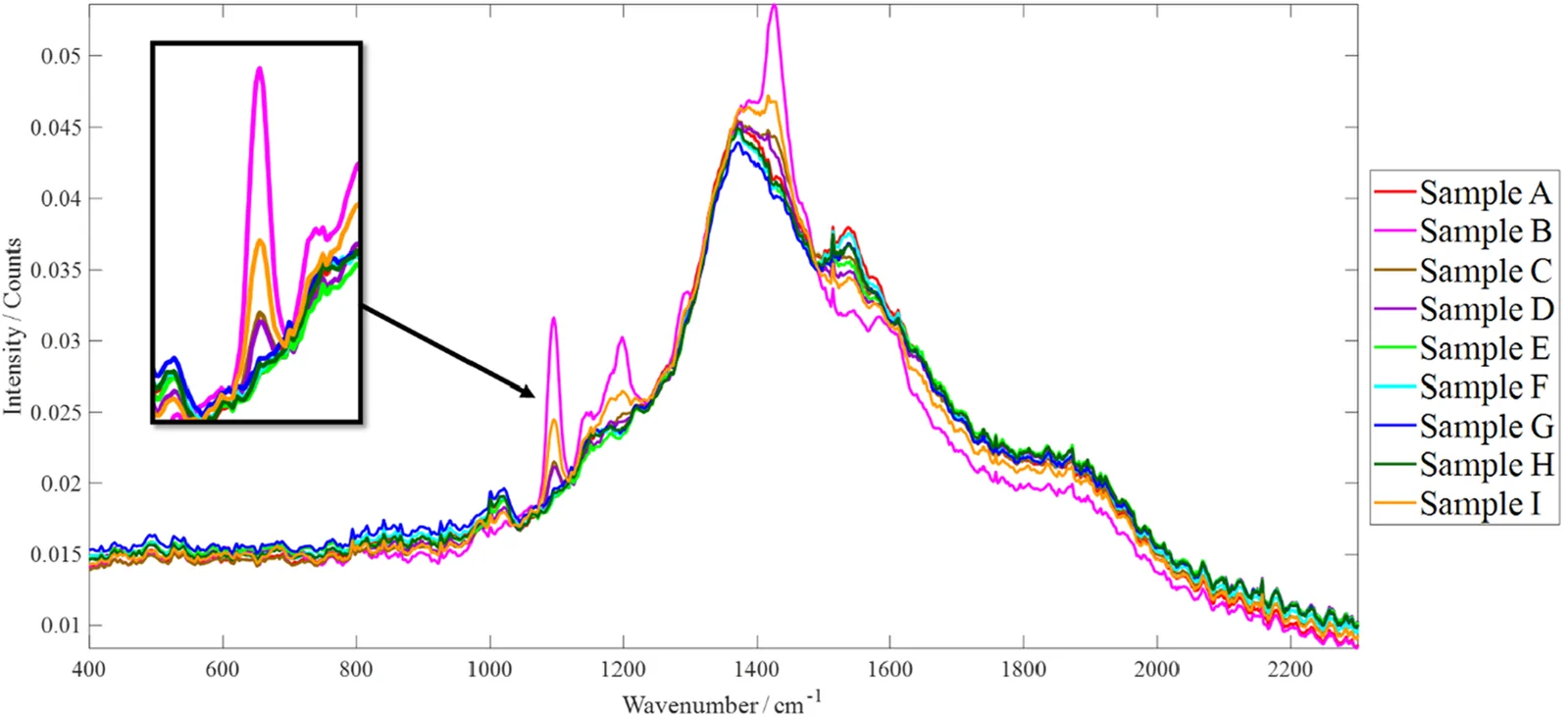
Normalized SERS spectra of samples A to I which were purchased from online retailers. A total of three spectra were recorded from each sample and then averaged.

### Univariate Analysis for the Prediction of Sudan IV Dye Concentration

Although visual inspection of spectra can provide an initial indication of Sudan IV dye adulteration through the presence and relative intensity of characteristic Raman bands, reliable authentication requires a quantitative approach. In particular, two key questions must be addressed: (i) whether the adulterant is present? and (ii) at what concentration? To enable quantitative assessment, a calibration model was constructed using the peak height at 1096 cm^–1^ and the known concentrations of Sudan IV dye spiked into palm oil (Figure 1). The resulting model (Figure 5) showed good model fit (*R* ^2^ = 0.9309), together with low root-mean-square error values for calibration (RMSEC = 0.0061 mg/mL), cross-validation (RMSECV = 0.0057 mg/mL), and prediction (RMSEP = 0.0059 mg/mL). A low limit of detection (LOD) of 0.0065 mg/mL was also achieved, demonstrating the suitability of the portable SERS approach for semiquantitative screening of Sudan IV adulteration.^27^

**Figure 5:**
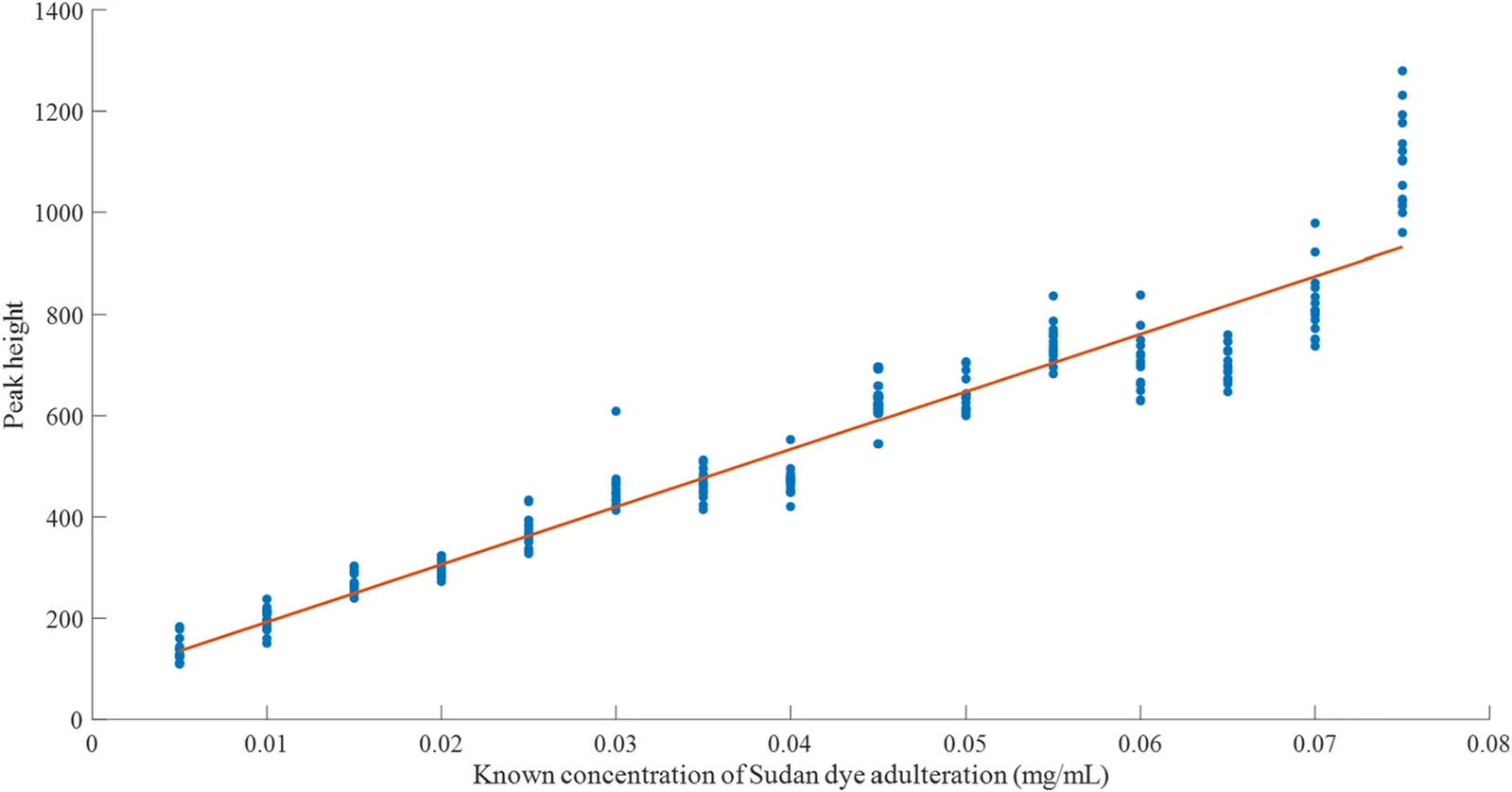
Univariate calibration model for predicting Sudan IV concentrations in all samples based on peak height of raw data versus known dye concentrations. In total, 5 samples from each concentration were put through extraction, followed by 3 SERS measurements for each of the 5 samples, which gives a total of 15 spectra per concentration. The highest concentration (0.075 mg/mL) exhibits higher variance, and this may be due to saturation of the nanoparticle surface.

To enable this workflow, citrate-stabilized gold nanoparticles, prepared using the method of Lee and Meisel, were employed as the SERS substrate. Although these nanoparticles offer lower intrinsic sensitivity compared to more advanced engineered substrates, such as the bimetallic silver-gold nanoflower structures reported by Adade *et al.*, they were selected on the basis of cost, simplicity, reproducibility, and suitability for field deployment. This choice directly supports the primary aim of the study, which is to evaluate whether a straightforward, commercially transferable SERS workflow combined with a hand-held Raman system can enable rapid screening of authentic palm oil samples collected directly from markets. Importantly, citrate-reduced gold nanoparticles can be synthesized rapidly without specialized equipment or complex fabrication steps, making them more practical for routine use in resource-limited settings where operational simplicity is critical.

Within this context, it is important to consider the performance of alternative analytical techniques previously reported for Sudan IV detection in food matrices. Methods such as partial-filling micellar electrokinetic chromatography (3.07 μg/mL), capillary electrophoresis (609.8 μg/L), and liquid chromatography–tandem mass spectrometry (down to 0.23 ng/mL) offer substantially lower limits of detection.^28–31^ However, these techniques typically require laboratory-bound instrumentation, extensive sample preparation, and longer analysis times, which limits their suitability for rapid or field-based screening applications.

The calibration model was subsequently applied to predict Sudan IV dye concentrations in unknown samples. The predicted concentrations of Sudan IV in the oils purchased online (Table 1), as determined by the univariate calibration model and LOD, indicate that oils B, C, D, and I contain measurable levels of the dye. The predicted concentrations of Sudan IV for the oils collected in Cameroon can be found in Supporting Information Table 1 and are all below the LOD for this dye.

**Table 1:** Predicted Concentrations of Sudan IV Dye (mg/mL) for Each Sample Bought Online, as Well as the Average and Standard Deviation

	predicted Sudan IV concentration (mg/mL, replicates)				
sample	1	2	3	average and (standard deviation)	(Sudan IV concentration above/below LOD (0.0065 mg/mL)
A	0.0003	0.0004	–0.0005	0.0001 (0.0005)	below
B	0.0543	0.0535	0.0549	0.0542 (0.0007)	above
C	0.0109	0.0116	0.0105	0.0110 (0.0006)	above
D	0.0081	0.0088	0.0086	0.0085 (0.0004)	above
E	0.0014	–0.0004	0.0006	0.0005 (0.0009)	below
F	0.0000	0.0006	–0.0002	0.0001 (0.0004)	below
G	0.0001	0.0007	–0.0004	0.0001 (0.0006)	below
H	0.0010	0.0017	0.0012	0.0013 (0.0004)	below
I	0.0244	0.0231	0.0244	0.0240 (0.0008)	above

A drawback of this model is that it predicts negative dye concentrations for a small number of samples. This arises from leverage effects in the calibration model, where points at the extremes of the calibration range exert greater influence on the regression fit. As a result, confidence intervals widen toward both ends of the calibration curve, increasing prediction uncertainty in these regions. Consequently, samples near zero concentration can yield slightly negative predicted values as a statistical artifact of the model rather than a physically meaningful result. In this case, the negatively predicted samples corresponded to those with no target analyte and therefore lay outside the calibrated concentration range, making such outputs not unexpected. Moreover, including zero concentration (blank) in the calibration model is not always appropriate, as it implicitly assumes a zero analytical response at zero concentration, which is rarely realistic in practice due to background signal and instrumental noise. Importantly, all findings were independently validated using LC-MS as an orthogonal analytical technique, confirming the reliability of the results.

### LC-MS Verification of Sudan IV Detection

Mass spectrometry is considered the gold standard for regulatory testing of Sudan IV, so both the Cameroon samples and those purchased online were analyzed using LC-MS in positive electrospray ionization (ESI) mode, along with in-line upstream UV/vis detection (which corroborated the MS signal via the presence of a visible 525 nm peak (dependent on Sudan IV concentration)). The LC-MS results clearly confirmed the presence of Sudan IV in samples B, C, D, and I (Figure 6). LC-MS also detected a very low level of Sudan IV in sample H; with a peak area substantially lower than in the spiked control and the other Sudan IV-positive oils. This latter observation contrasts with the SERS measurements for which sample H showed no detectable dye, likely due to the concentration being below the LOD for this method; although on closer inspection of Table 1, sample H had the highest predicted concentration of all the samples below the LOD threshold.

**Figure 6:**
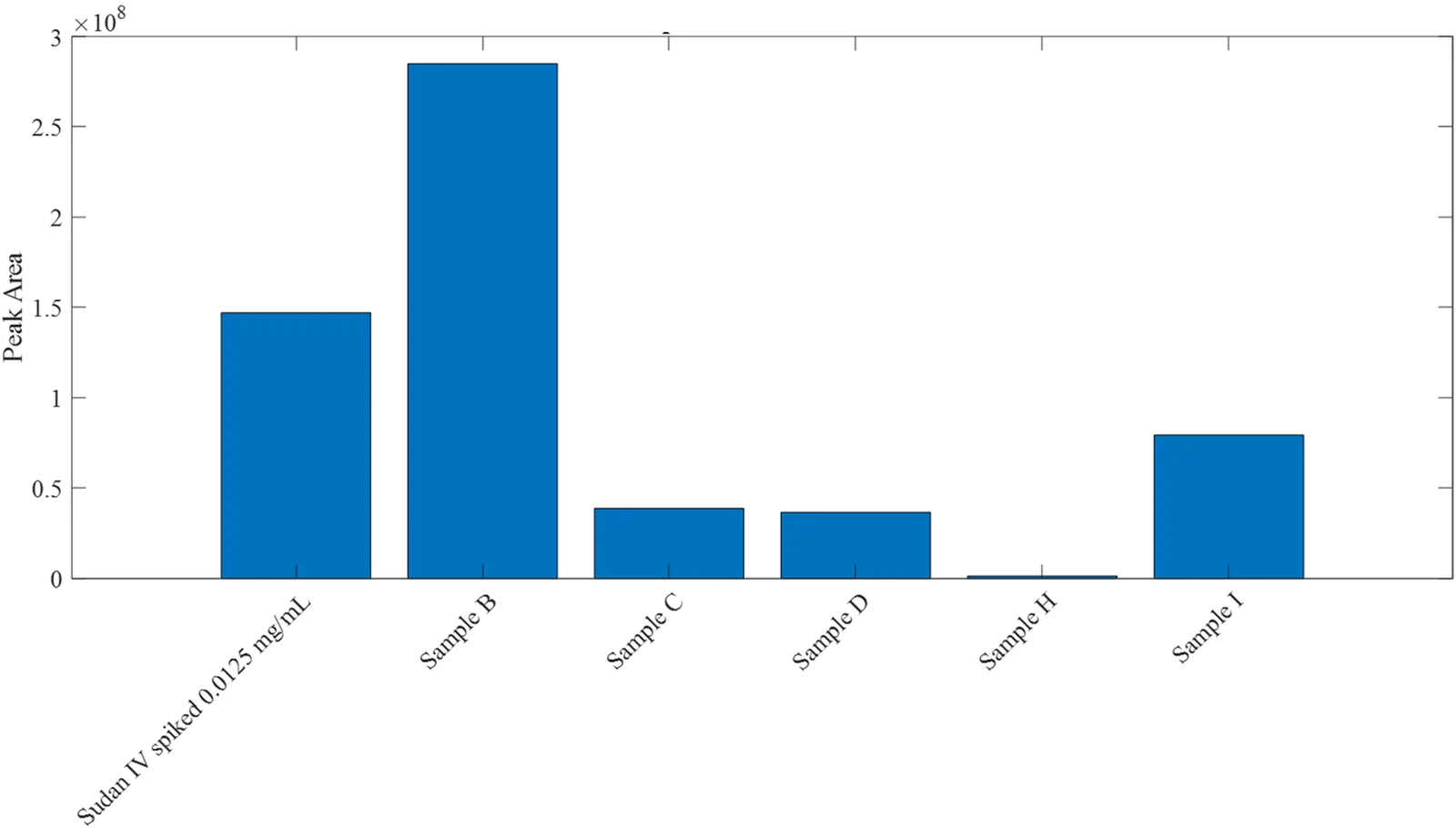
Extracted single ion chromatogram peak areas ([MH]^**+**^ = 381) for samples with suspected Sudan IV contamination. Blank samples were analyzed under identical experimental conditions, and no detectable peaks corresponding to Sudan IV were observed (data not shown). Samples were run on the LC-MS system as previously described and plotted using Thermo Scientific’s Freestyle application within their Xcalibur software.

Combining SERS with data analysis techniques – including PCA for exploratory analysis and univariate methods for quantification – enabled the detection of Sudan IV in four oils purchased online. Despite suggestions that palm oils from Africa may be adulterated with Sudan IV, making these oils highly vibrant in color, the 64 oils sourced from Cameroon were devoid of this hydrophobic azo dye. Subsequent LC-MS analysis corroborated these findings while also detecting trace Sudan IV in a fifth oil purchased online at concentrations below the SERS limit of detection (0.0065 mg/mL).

## Conclusions

Sudan IV adulteration is widely reported across West African countries; however, none of the palm oil samples sourced from Cameroon in this study tested positive for the dye. In contrast, portable SERS identified four of nine samples purchased from an online UK retailer as adulterated, with the presence of Sudan IV subsequently confirmed by LC-MS. LC-MS analysis also detected Sudan IV in a fifth sample, although at a concentration below the SERS LOD. These findings suggest that adulteration may not occur at the point of origin but could instead be introduced further along the supply chain, highlighting the importance of monitoring not only production sites but also downstream distribution and retail channels. This creates a need for mobile-capable guardians that can provide rapid, in-field screening at multiple points throughout the supply chain, enabling potentially adulterated products to be identified before reaching consumers. In this context, portable SERS offers clear practical advantages, being rapid, compact, and requiring minimal sample preparation, making it well suited for deployment as a mobile screening tool without the specialized laboratory infrastructure required for LC-MS analysis.

Future work should prioritize enhancing analytical sensitivity through the use of more modern hand-held Raman spectrometers, improved validation using a larger and more diverse set of commercial samples, alongside interlaboratory comparison studies, as has started by the SERS community.^32^ These activities will be essential to demonstrate robustness, ensure reproducibility, and facilitate regulatory acceptance for deployment in field-based screening programs.

## Supplementary Material



## Data Availability

The data are available on request. Data processing algorithms are available via: https://github.com/Biospec/.

## Notes

SEM images of citrate-reduced gold nanoparticles (Figure S1); ζ-potential distribution of citrate-reduced gold nanoparticles measured using the Malvern Zetasizer Nano ZS (Malvern Panalytical, Malvern, UK) fitted with a 532 nm laser (Figure S2); results from a Zetasizer of the average hydrodynamic diameter of citrate-reduced gold nanoparticles measured using the Malvern Zetasizer Nano ZS (Malvern Panalytical, Malvern, UK) fitted with a 532 nm laser (Figure S3); Raman and SERS spectra of negative and positive control samples are shown for the glass vial, citrate-reduced gold nanoparticles, aggregated nanoparticles (water + NaCl), and extracted palm oil samples spiked with Sudan IV at three concentrations, including a dye-free control (Figure S4); image of three palm oil samples from the study, highlighting the variation in colour between samples (Figure S5); SERS spectra of Sudan IV spiked in methanol, illustrating detector saturation and consequent clipping of Raman peak intensities (Figure S6); predicted concentrations from SERS analysis of Sudan IV dye (mg/mL) for each sample from Cameroon, as well as the average and standard deviation (Table S1) (PDF)
