# Supplementary material for: No Time to Dye: Portable Surface-Enhanced Raman Scattering for On-Site Detection of Sudan IV Dye in Palm Oil
Source: ACS Omega. 2026 Oct 8;11(42):65011–9. doi: 10.1021/acsomega.6c06638 (PMC13718203; doi:10.1021/acsomega.6c06638)
Supplement: Supplementary file 1 [file ao6c06638_si_001.pdf]

No Time to Dye: Portable Surface Enhanced Raman Scattering for On-site Detection of Sudan IV Dye in Palm Oil

Joe Stradling[1], Amy Colleran[1], Nigel Gotts[1], Yun Xu[1], James Simons[1], Wilfred A. Abia[2,3], Howbeer Muhamadali[1], Royston Goodacre*[1]

[1] *Centre for Metabolomics Research, Department of Biochemistry, Cell and Systems Biology, Institute of Systems, Molecular and Integrative Biology, University of Liverpool, Liverpool L69 7ZB, United Kingdom*

[2]*Institute for Global Food Security, School of Biological Sciences, Queens University Belfast, 19 Chlorine Gardens, Belfast BT9 5DL, Northern Ireland, UK*

[3] *Laboratory of Pharmacology and Toxicology, Department of Biochemistry, Faculty of Science, University of Yaounde 1, AEFAS 812, Yaounde, Cameroon*

*Corresponding author: roy.goodacre@liverpool.ac.uk

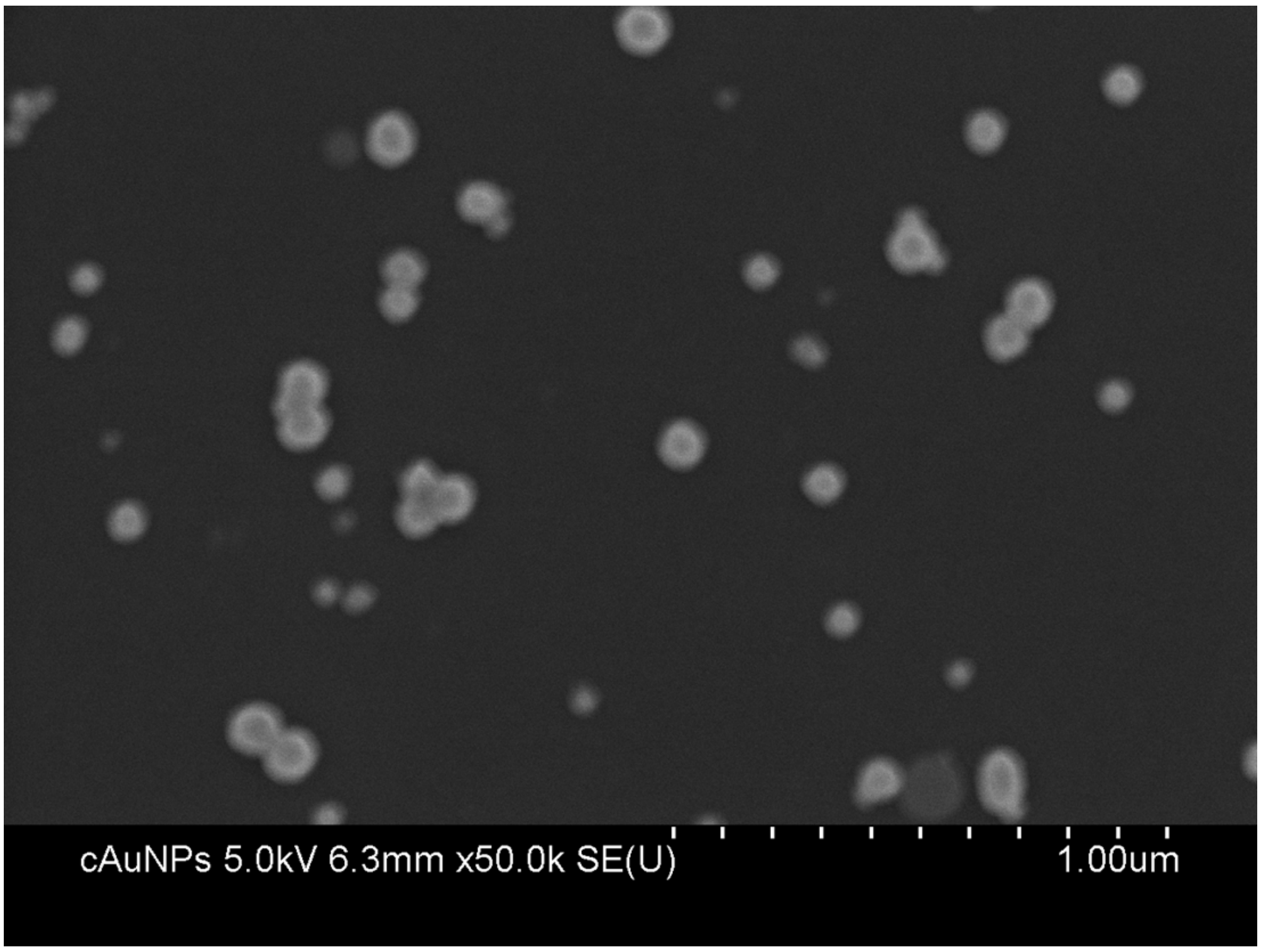

Supporting Information: Figure S1. SEM images of citrate-reduced gold nanoparticles. The nanoparticles were deposited on to Si wafers and imaged using a Hitachi S-4800 SEM.

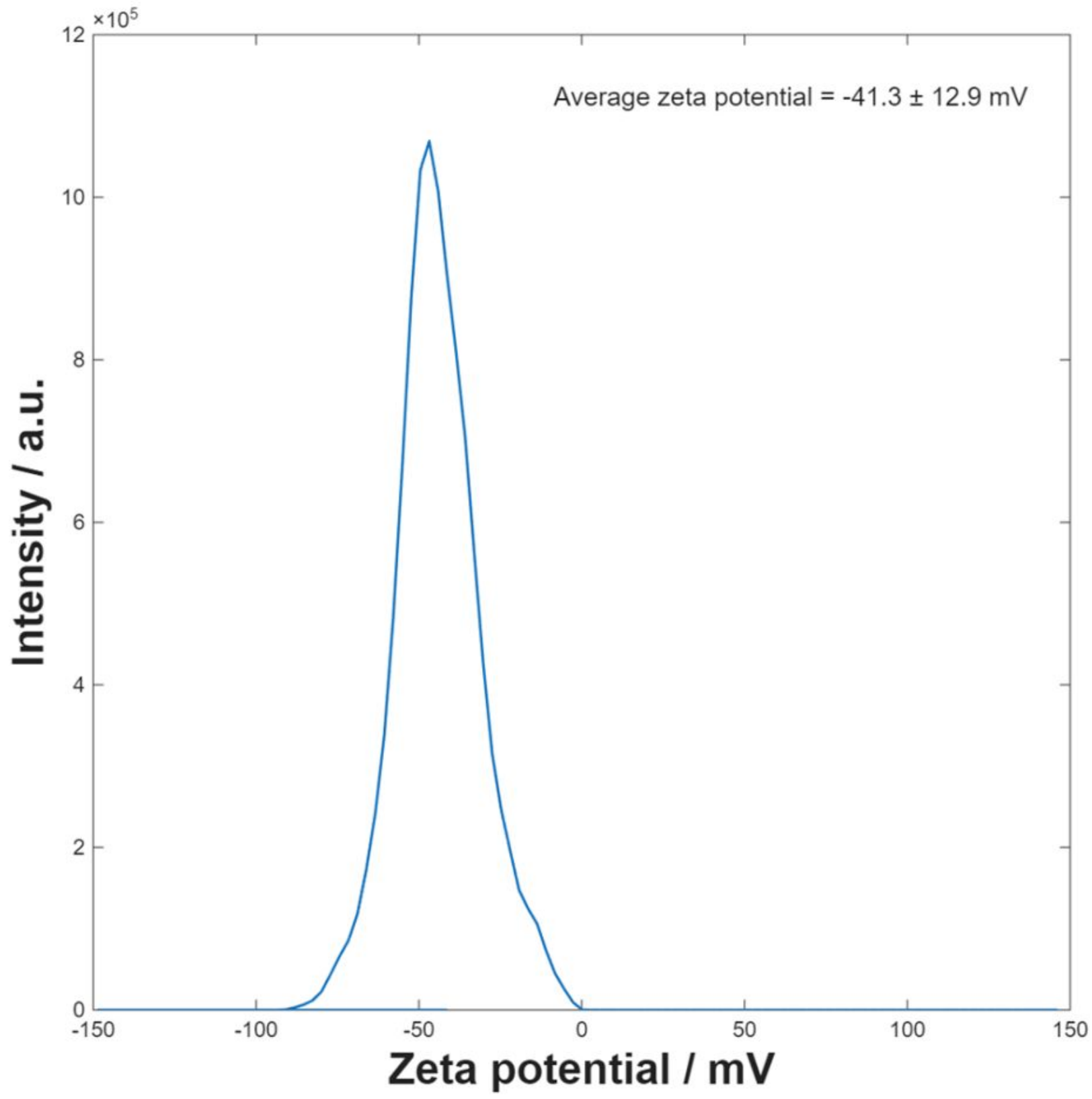

Supporting Information: Figure S2. Zeta potential distribution of citrate-reduced gold nanoparticles measured using the Malvern Zetasizer Nano ZS (Malvern Panalytical, Malvern, UK) fitted with a 532 nm laser.

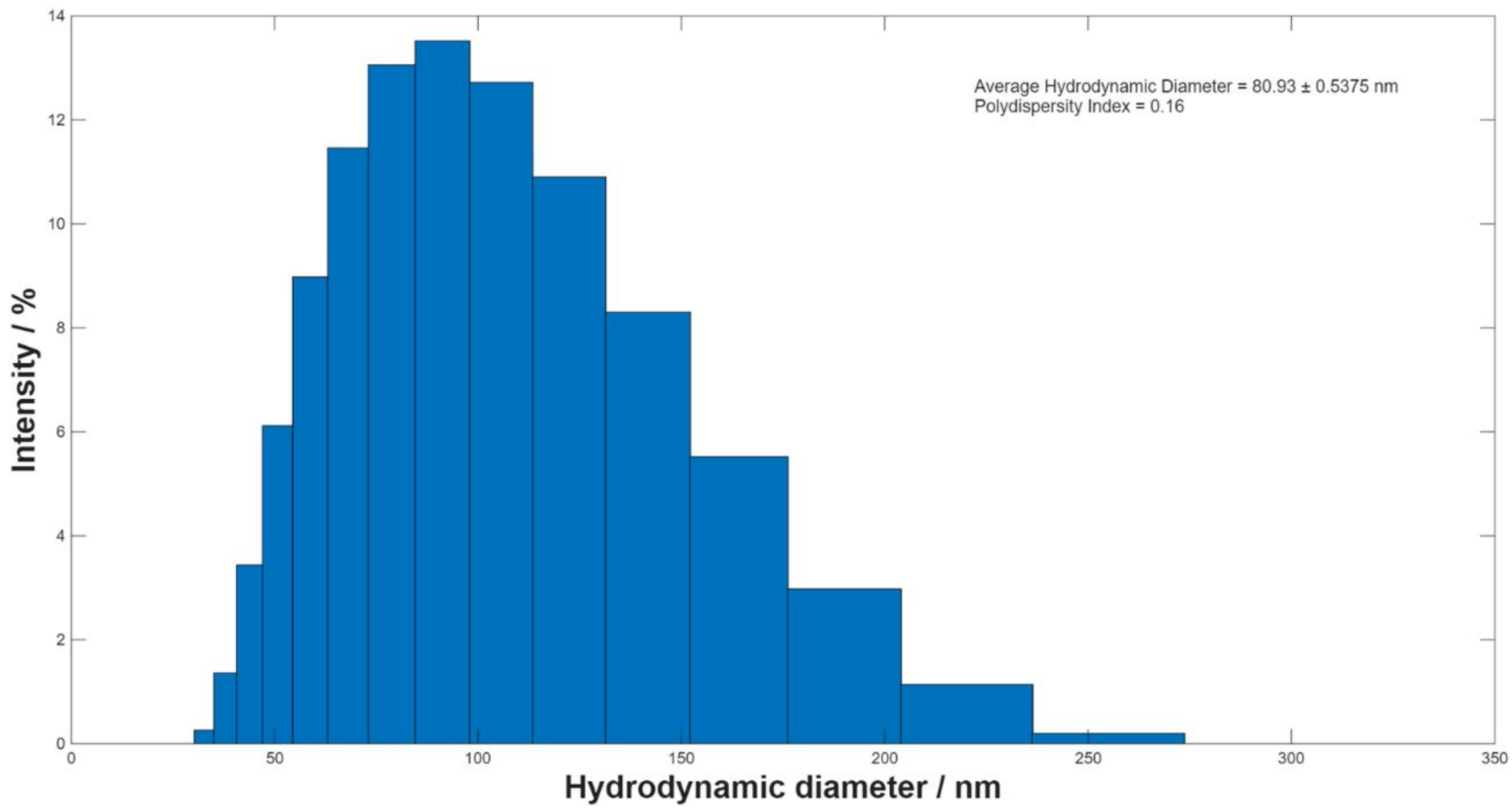

Supporting Information: Figure S3. Results from a Zetasizer of the average hydrodynamic diameter of citrate-reduced gold nanoparticles measured using the Malvern Zetasizer Nano ZS (Malvern Panalytical, Malvern, UK) fitted with a 532 nm laser. Histograms display size distribution from intensity**.**

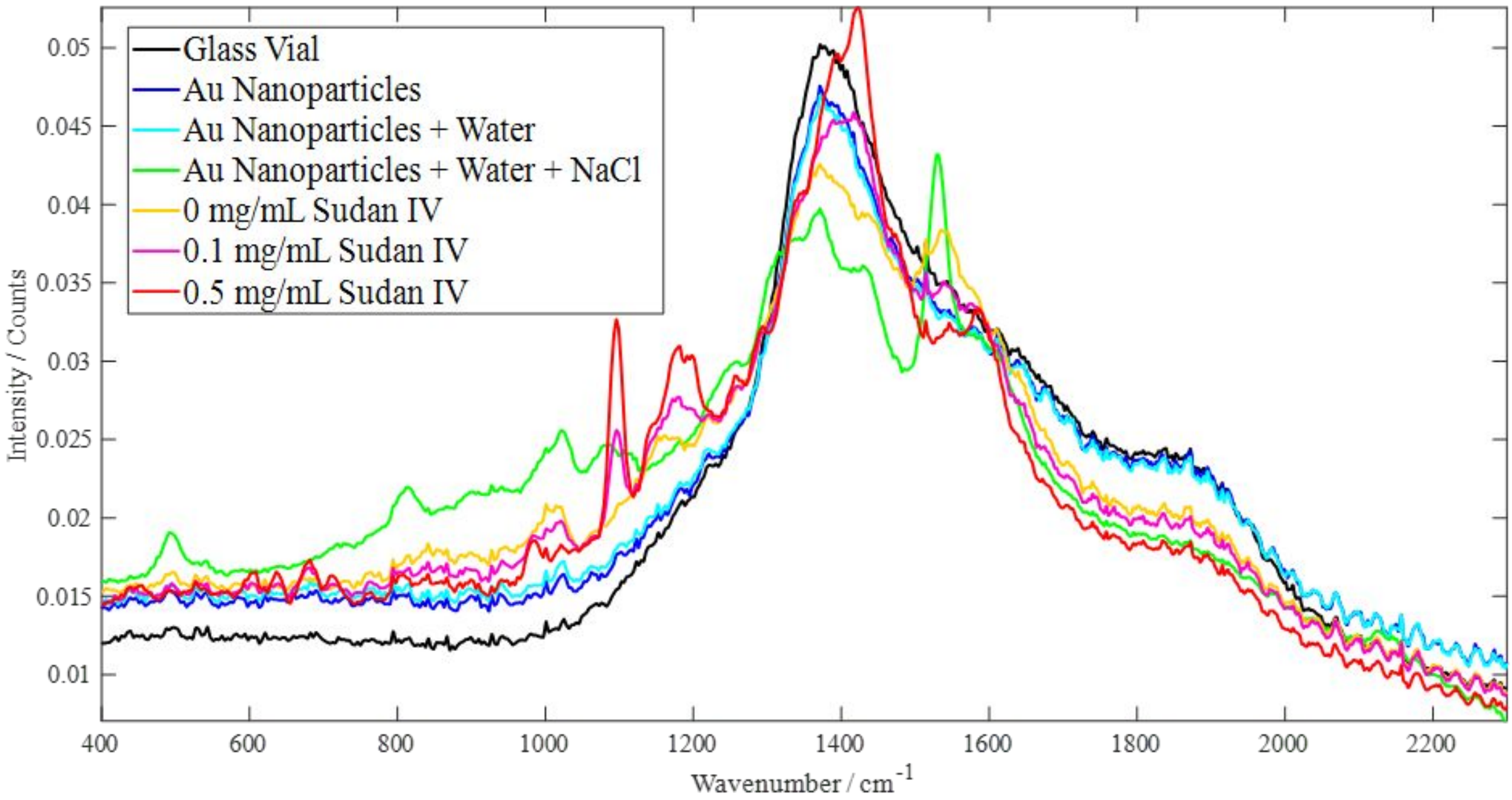

Supporting Information: Figure S4. Raman and SERS spectra of negative and positive control samples are shown for the glass vial, citrate-reduced gold nanoparticles, aggregated nanoparticles (water + NaCl), and extracted palm oil samples spiked with Sudan IV at three concentrations, including a dye-free control. The spectra of the 3 different levels of spiked Sudan dye were taken after extraction and drying.

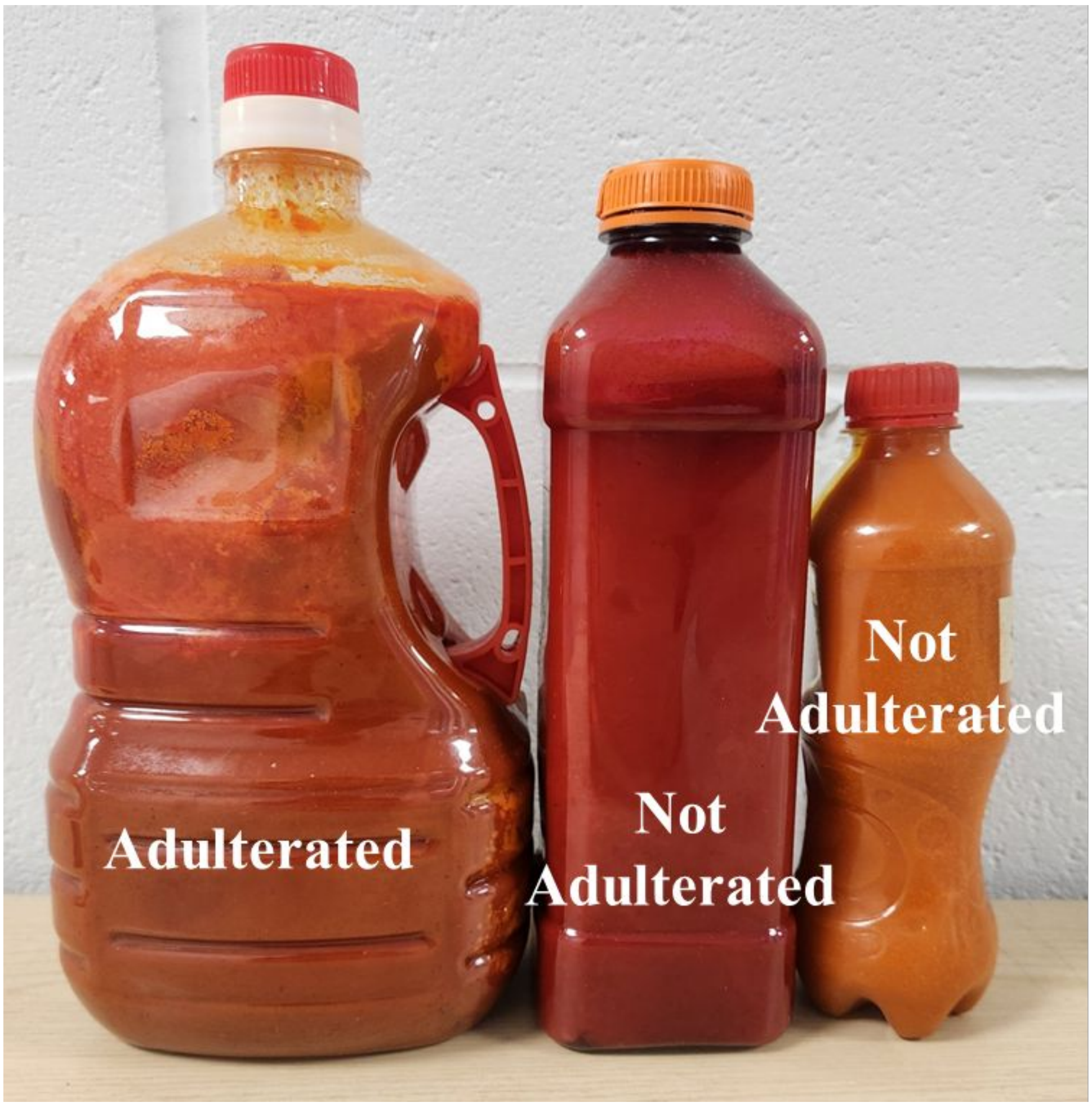

Supporting Information: Figure S5. Image of three palm oil samples from the study, highlighting the variation in colour between samples.

Palm oil naturally ranges in colour from pale orange to deep red, a variation largely driven by its differing levels of β-carotene. This natural variability means that two legitimate, unadulterated oils can look strikingly different from one another. When colourant dyes such as Sudan IV are added, they generally intensify the redness of the oil. Yet the effect is not always dramatic: low levels of added dye may shift the appearance only slightly - from light orange toward a somewhat deeper hue - making visual inspection unreliable. Complicating matters further, an oil that is naturally rich in β-carotene may appear redder than an oil that has been adulterated but contains relatively little dye. These overlapping colour ranges highlight the limitations of colour-based assessments and underscore the need for analytical techniques capable of reliably detecting the presence of Sudan dyes in palm oil. As shown, even though the sample on the left has been adulterated, it still presents a paler appearance compared with the middle sample. This highlights the overlap between natural colour variability and dye-induced colour changes.

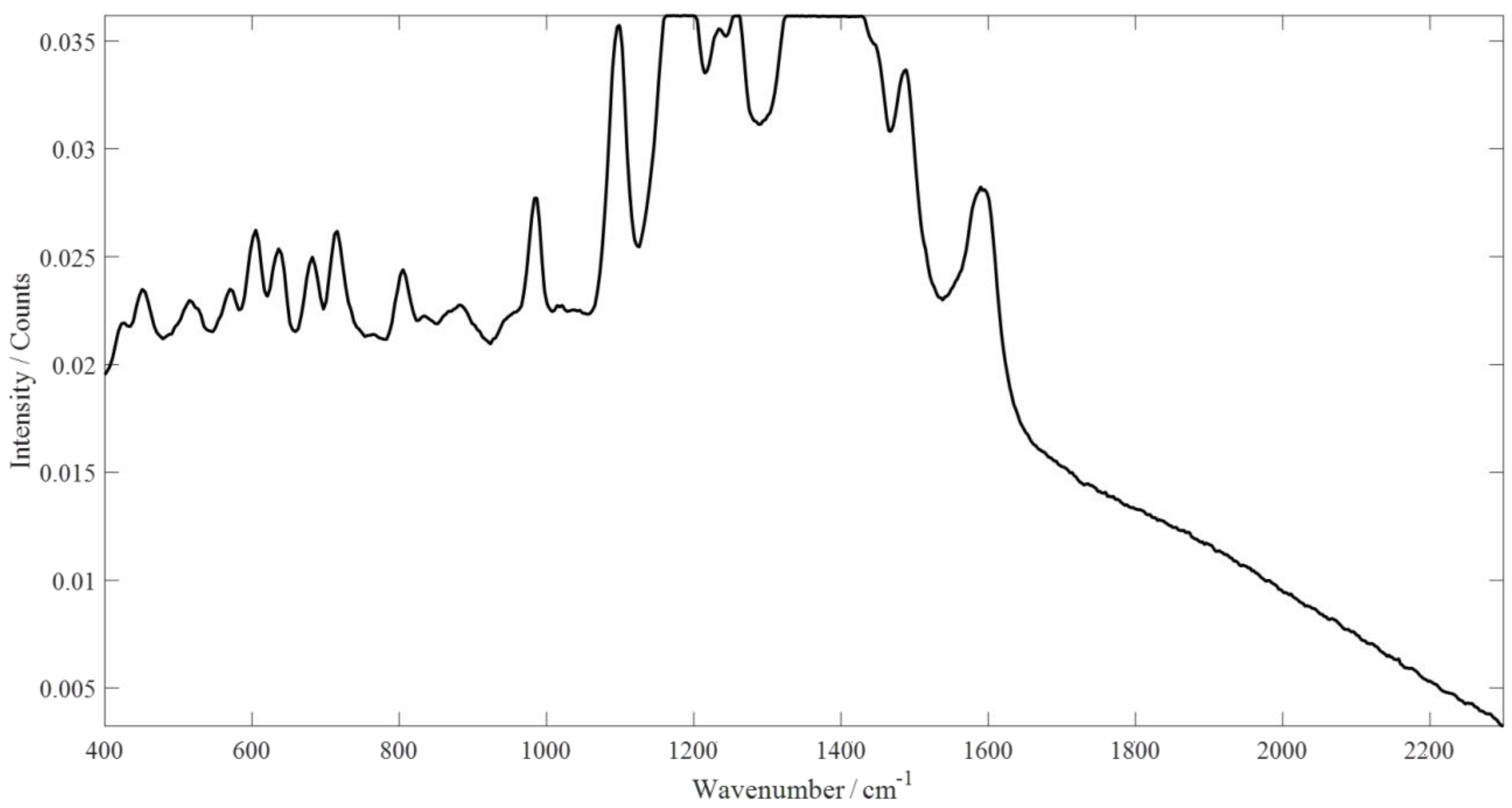

Supporting Information: Figure S6. SERS spectra of Sudan IV spiked in methanol, illustrating detector saturation and consequent clipping of Raman peak intensities.

Table S1. Predicted concentrations from SERS analysis of Sudan IV dye (mg/mL) for each sample from Cameroon, as well as the average and standard deviation.

Sample	Predicted Sudan IV Concentration (mg/mL, replicates)				(Sudan IV concentration above/below LOD (0.0065 mg/mL)
	1	2	3	Average and (standard deviation)	
1	0.0009	0.0012	0.0009	0.0010 (0.0002)	Below
2	0.0006	0.0009	0.0006	0.0007 (0.0002)	Below
3	0.0011	0.0005	0.0021	0.0012 (0.0008)	Below
4	0.0003	0.0004	0.0012	0.0006 (0.0005)	Below
5	0.0012	0.0008	0.0013	0.0011 (0.0003)	Below
6	0.0005	0.0007	0.0015	0.0009 (0.0006)	Below
7	-0.0006	0.0004	-0.0004	–0.0002 (0.0005)	Below

8	0.0006	0.0011	0.0013	0.0010 (0.0004)	Below
9	0.0020	0.0014	0.0008	0.0014 (0.0006)	Below
10	0.0012	0.0005	0.0011	0.0009 (0.0004)	Below
11	0.0014	0.0015	0.0015	0.0015 (0.0001)	Below
12	0.0005	0.0010	0.0013	0.0009 (0.0004)	Below
13	0.0013	0.0006	0.0008	0.0009 (0.0004)	Below
14	0.0001	0.0021	0.0000	0.0007 (0.0013)	Below
15	0.0012	0.0004	0.0011	0.0009 (0.0005)	Below
16	0.0003	0.0013	0.0013	0.0010 (0.0006)	Below
17	0.0010	0.0010	0.0007	0.0009 (0.0002)	Below
18	0.0004	0.0015	0.0008	0.0009 (0.0006)	Below
19	0.0012	0.0012	0.0010	0.0011 (0.0001)	Below
20	0.0006	0.0017	0.0011	0.0011 (0.0006)	Below
21	0.0005	0.0019	0.0008	0.0011 (0.0008)	Below
22	0.0005	0.0008	0.0011	0.0008 (0.0003)	Below
23	0.0023	0.0015	0.0004	0.0014 (0.0010)	Below
24	0.0000	0.0005	0.0004	0.0003 (0.0003)	Below
25	0.0012	−0.0001	0.0011	0.0007 (0.0008)	Below
26	0.0012	0.0010	0.0008	0.0010 (0.0002)	Below

27	0.0011	0.0010	0.0017	0.0013 (0.0004)	Below
28	0.0008	0.0004	0.0021	0.0011 (0.0009)	Below
29	0.0021	0.0018	0.0013	0.0017 (0.0004)	Below
30	0.0004	0.0011	0.0006	0.0007 (0.0004)	Below
31	0.0010	0.0014	0.0014	0.0010 (0.0004)	Below
32	0.0010	0.0006	0.0006	0.0007 (0.0002)	Below
33	0.0003	0.0006	0.0006	0.0005 (0.0002)	Below
34	0.0010	0.0019	0.0007	0.0012 (0.0006)	Below
35	0.0008	-0.0003	0.0013	0.0006 (0.0008)	Below
36	0.0009	0.0007	0.0007	0.0008 (0.0001)	Below
37	-0.0002	0.0007	0.0001	0.0002 (0.0005)	Below
38	0.0007	0.0004	0.0007	0.0006 (0.0002)	Below
39	0.0005	0.0007	0.0005	0.0006 (0.0001)	Below
40	-0.0007	0.0014	0.0004	0.0004 (0.0011)	Below
41	0.0009	0.0005	0.0009	0.0008 (0.0003)	Below
42	0.0006	0.0017	0.0019	0.0014 (0.0007)	Below
43	0.0005	0.0011	0.0006	0.0007 (0.0003)	Below
44	-0.0002	0.0002	-0.0005	-0.0002 (0.0004)	Below
45	0.0014	0.0004	0.0005	0.0008 (0.0005)	Below

46	0.0019	0.0005	0.0006	0.0010 (0.0008)	Below
47	0.0009	0.0011	0.0014	0.0011 (0.0003)	Below
48	0.0011	0.0017	0.0013	0.0014 (0.0003)	Below
49	0.0011	0.0008	0.0017	0.0012 (0.0005)	Below
50	0.0005	0.0009	0.0014	0.0009 (0.0005)	Below
51	0.0004	0.0003	0.0004	0.0004 (0.0001)	Below
52	0.0016	0.0015	0.0013	0.0015 (0.0001)	Below
53	0.0017	0.0019	0.0013	0.0016 (0.0003)	Below
54	0.0008	-0.0008	0.0001	0.0000 (0.0008)	Below
55	0.0004	0.0010	0.0008	0.0011 (0.0003)	Below
56	0.0023	0.0012	0.0009	0.0015 (0.0008)	Below
57	0.0012	0.0002	-0.0001	0.0004 (0.0007)	Below
58	0.0008	0.0001	0.0008	0.0006 (0.0004)	Below
59	0.0006	0.0005	0.0007	0.0006 (0.0001)	Below
60	0.0005	0.0011	0.0003	0.0006 (0.0004)	Below
61	0.0003	0.0016	0.0016	0.0012 (0.0008)	Below
62	0.0004	0.0013	0.0018	0.0012 (0.0007)	Below
63	0.0008	0.0009	0.0002	0.0006 (0.0004)	Below
64	0.0014	0.0005	0.0008	0.0009 (0.0005)	Below
